# Transdermal Delivery of Drugs with Microneedles—Potential and Challenges

**DOI:** 10.3390/pharmaceutics7030090

**Published:** 2015-06-29

**Authors:** Kevin Ita

**Affiliations:** College of Pharmacy, Touro University, Mare Island-Vallejo, CA 94592, USA; E-Mail: kevin.ita@tu.edu; Tel.: +1-707-638-5994; Fax: +1-707-638-5266

**Keywords:** transdermal, microneedles, drug delivery, dissolving microneedles, hydrogel-forming microneedles

## Abstract

Transdermal drug delivery offers a number of advantages including improved patient compliance, sustained release, avoidance of gastric irritation, as well as elimination of pre-systemic first-pass effect. However, only few medications can be delivered through the transdermal route in therapeutic amounts. Microneedles can be used to enhance transdermal drug delivery. In this review, different types of microneedles are described and their methods of fabrication highlighted. Microneedles can be fabricated in different forms: hollow, solid, and dissolving. There are also hydrogel-forming microneedles. A special attention is paid to hydrogel-forming microneedles. These are innovative microneedles which do not contain drugs but imbibe interstitial fluid to form continuous conduits between dermal microcirculation and an attached patch-type reservoir. Several microneedles approved by regulatory authorities for clinical use are also examined. The last part of this review discusses concerns and challenges regarding microneedle use.

## 1. Introduction

Hypodermic needles are used in clinical practice to deliver medications across the skin into the bloodstream. Injections with hypodermic needles are important from a clinical standpoint, but painful. They may also induce hypersensitivity; bruising, discomfort and bleeding at the site of administration, and in some cases are associated with risks of contamination. There are other concerns linked to their use including accidental needle stick injury and the necessity to train medical staff regarding the proper use of needles [1]. The difficulty in crossing the skin is caused by its anatomical peculiarities. The skin is the largest organ in the body. It is about 1.5 m^2^ in adults and provides protection for internal organs [1]. It also protects the human body against ingress of toxic chemicals and egress of water and other essential endogenous substances. Despite the large surface area of the skin, it is challenging for compounds including drugs and vaccines to cross the skin in therapeutically relevant amounts. The major barrier in the skin is provided by the stratum corneum (SC), which is the outermost layer of the skin [2]. The SC is 10–15 μm thick with 15–20 corneocyte layers. It is made up of corneocytes embedded in an intercellular lipid matrix. The main lipid classes in human SC are free fatty acids (FFAs), ceramides (CERs) and cholesterol which form two lamellar phases. These include the short periodicity phase (SPP) and the long periodicity phase (LPP) with repeat distances of approximately 6 and 13 nm, respectively [3]. Within the lipid lamellae, the lipids are mainly organized in a dense orthorhombic packing, although a fraction of lipids adopt a hexagonal packing [4]. Below the stratum corneum is the viable epidermis (VE), which is a cellular, avascular tissue measuring 50–100 μm thick [5]. The VE consists mainly of keratinocytes and approximately 40% protein, 40% water and 15%–20% lipids [6]. The undulating epidermal–dermal junction consists of papilla that project into the dermis [6]. Cells in the basal layer of the epidermis form the most important structural and functional connection to the dermis below [6]. The stratum corneum and viable epidermis together form the full epidermis. There is a basement membrane at the base of the epidermis and the existence of tight junctions in the viable epidermis has been recently documented. Base membrane and tight junctions may both offer resistance to the transport of molecules across the epidermis [5]. Andrews *et al.* recently reported that techniques that only permeabilize stratum corneum are much less effective than those that increase the permeability of the entire epidermis [5] The authors showed that removal of stratum corneum dramatically increased skin permeability, but removal of the full epidermis increased skin permeability by another 1–2 orders of magnitude [5]. The dermis is the thickest component of the skin, up to 4 mm in depth. Its upper layer, the 100–200 μm thick papillary dermis, consists of thin collagen bundles, elastin fibers, fibrocytes. There is also water, electrolytes, plasma proteins and polysaccharides–polypeptide complexes. Below this layer is the reticular dermis, made up of thick collagen bundles and coarse elastic fibers [6]. Although the stratum corneum (SC) is the major contributor to the barrier properties of the skin, the role of the full epidermis should be taken into consideration [5,7]. Only compounds which are able to get into the SC, diffuse through living epidermis and pass through the upper part of the papillary dermis has the potential to reach circulation and exhibit systemic effects [7]. The dermis contains blood vessels, lymphatics and nerves, as well as the various skin appendages. Below the reticular dermis lies the hypodermis (subcutaneous fat tissue), which may have a thickness of up to several millimeters. Comprising fat microlobules and fibrous collagen, it also contains blood vessels, lymphatics and nerves [6].

**Figure 1:**
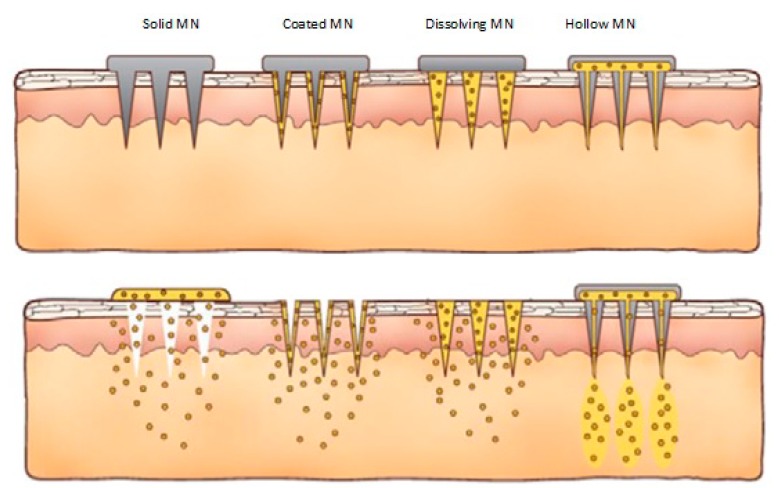
Different types of microneedles: solid, coated, dissolving and hollow (Reprinted from [14] with permission. Copyright 2012 Elsevier).

Microneedles (MN) are currently being utilized to enhance transdermal delivery of small and large molecules. With the emergence of microfabrication manufacturing technology over the past several decades, MN have been developed by academic laboratories and pharmaceutical companies [8,9,10,11,12,13]. Transdermal MNs create micron sized pores in the skin to enhance delivery of the drug across the skin [11,13]. MN are ideal for patient adherence as they do not stimulate nerves that are associated with pain. MN improve patient compliance as patient with needle phobia will be more likely to apply the patch because of its painlessness [13]. Additionally, patients can administer the drug by themselves [13]. When MN are fabricated in arrays on a backing that can be applied to the skin like a bandage, the device is called a MN patch [8]. MN can be divided into four categories (Figure 1): hollow, solid, coated and polymer [6]. Hollow MN are like regular hypodermic needles but shorter in length. A liquid formulation of the drug is infused through bores in the MN. Solid MN are used to create holes in the skin. Subsequently a patch is then applied. Coated MN are MN coated with the drug while polymer MN are made from polymers that can be dissolving, nondissolving or hydrogel-forming.

## 2. Hollow Microneedles

Hollow microneedles (HM) can be fabricated from a commercially available 30 gauge hypodermic needles [15]. Pressure, and thereby flow rate, can be changed in HM for a rapid bolus injection, a slow infusion or a varied delivery rate [14]. HM can also be used to administer a larger dose of drug solution [16]. Verbaan *et al.* fabricated HM from 30G stainless steel needles [15]. The 4 × 4 pattern of holes was drilled in a polyetheretherketone mold (diameter 9 mm). Then, the needles were placed through the holes at a predetermined length of 300, 550, 700 and 900 μm. Subsequently, the needles were cut and glued at the back of the mold. A manual applicator was also designed for the MN array [15].

Aoyagi *et al.* fabricated long thin holes with a high aspect ratio of 100 (diameter: 10 μm, depth: 1 mm) in biodegradable polymer material using a UV excimer laser [17]. Holes having diameters of 10, 20 and 50 μm were fabricated from the side surface of a polylactic acid (PLA) sheet. The laser fabrication approach was then applied to a PLA microneedle, which was fabricated by a micromolding technique. A hole was fabricated along the centerline of a microneedle using the above-mentioned approach and it was confirmed experimentally that blood plasma was taken into the hole by capillary force [17].

There is also a report in the literature on a novel method for fabricating HM using a composite of vertically-aligned carbon nanotubes and polyimide [18]. Patterned bundles of carbon nanotubes were used as a porous scaffold for defining the MN geometry. Polyimide resin was then wicked through the carbon nanotube scaffold to reinforce the structure and provide the required mechanical strength for achieving skin penetration. The high aspect ratio and bottom-up assembly of carbon nanotubes makes it possible to create MN in a single step of nanotube fabrication [18].

For HM, it is important that adequate and constant flow rate is maintained for transdermal drug delivery, without compromising the mechanical strength of the needles. The main factor affecting the flow rate is the compression of the dense dermal tissue at the needle tip during insertion [19]. Sometimes, it is advantageous to have very sharp MN with the bore of the MN offcentered or on the side of the microneedle. Increasing microneedle bore may increase the flow rate; however, this results in a decreased MN strength and a reduction in sharpness. Another way to increase the MN strength is by applying a metal coating on the MN. However, this may decrease MN sharpness [19]. Hollow out-of-plane silicon microneedles were fabricated. A sequence of deep-reactive ion etching (DRIE), anisotropic wet etching and conformal thin film deposition was used. Tip curvature was defined by lithography and the length of the needles varied between 150 and 350 micrometers [20]. To fabricate hollow microneedles, Pérennès first fabricated a master through a double deep X-ray lithography process [21]. First, a polymethylmethacrylate (PMMA) sheet was exposed to produce single PMMA parts with a sawtooth profile. The tip angle of each tooth determined the final tip angle of the microneedles. The PMMA parts were then assembled and glued on a conductive substrate and then exposed through a second X-ray mask containing an array of hollow triangles as absorbing structures [21]. A metal layer was then electrodeposited around the needles in order to form the future base of the array. A polyvinyl alcohol (PVA) solution was cast on top of the master to form a negative mold of the MN array after low temperature curing and peel-off steps. A liquid PMMA solution was then cast on top of the PVA negative mold and after the full PMMA polymerization, the PVA was dissolved in water [21].

Silicon MN are justified by their mechanical properties and their biocompatibility potential. However inconveniences such as high production costs or fragility have spurred researchers to look for other options [1].

Hollow silicon MN were fabricated by using isotropic etching followed by anisotropic etching to obtain a tapered tip [22]. Silicon MN of 300 μm in height, with 130 μm outer diameter and 110 μm inner diameter at the tip followed by 80 μm inner diameter and 160 μm outer diameter at the base were fabricated using this technique. In order to improve the biocompatibility of MN, the fabricated microneedles were coated with titanium (500 nm) by sputtering technique followed by gold coating using electroplating. A breaking force of 225 N was obtained for the fabricated MN, which is 10 times higher than the skin resistive force.

Hollow microneedles can also be fabricated using other micro-electro-mechanical systems (MEMS) technologies such as laser micromachining, deep reactive ion etching, integrated lithographic molding technique, wet chemical etching and X-ray photolithography [14].

AdminPen^®^ MN have also been fabricated [17]. These are hollow stainless steel MN of varying lengths from 600 to 1500 µm, which can be connected to a syringe and used to deliver liquid formulations. AdminStamp^®^ devices contain AdminPatch^®^ microneedle arrays attached to an applicator with six stainless steel screws. They can also be used to porate the skin without any liquid. When used in this way, they are like solid MN because they first create the holes before a drug solution is applied.

Hollow microneedles can deposit a compound directly into the viable epidermis or the dermis avoiding the stratum corneum. This is especially useful for the delivery of high molecular weight compounds such as proteins, oligonucleotides and vaccines. Transdermal delivery of insulin continues to represent a significant scientific challenge. Cheung *et al.* used 1100 and 1400 µm long stainless steel microneedles to deliver insulin across porcine skin [12]. Two commercial MN patches, namely, AdminPatch Array 1200 MN and AdminPatch Array 1500 MN array (AdminMed, Sunnyvale, CA, USA) were used to pretreat the pig skin. The authors investigated the effect of insertion force and found out that when porcine skin was pretreated with an applied force of 60.5 and 69.1 N, the resultant amount of insulin permeated was approximately 3 and 25 µg over a four-hour period [12].

A hollow microneedle was used by Jun *et al.* to deliver a phenylephrine (PE) solution into the anal sphincter muscle as a method for treating fecal incontinence [23]. The goal of the study was the local targeted delivery of phenylephrine into the sphincter muscle through the perianal skin with minimal pain, resulting in the increase of resting anal sphincter pressure. PE was administered on the left and the right sides of the anus of a rat through the perianal skin using 1.5 mm long hollow microneedle [23].

Specific and effective gene silencing of certain diseases can be achieved by an accurate knowledge of the target mRNA sequence and rational design of its complementary antisense agents for protein downregulation. Antisense technology uses agents like antisense oligonucleotides, ribozymes, short interfering RNA (siRNA), microRNA (miRNA), apatamers, and others to manipulate gene expression and treat various pathological disorders [24]. Luo *et al.* used a combination of microneedle (MN) arrays and biochemical approaches for localized intratissue delivery of oligonucleotides in three-dimensional tissue models [25]. This approach is applicable to transdermal drug delivery.

It is desirable that hollow microneedles possess adequate mechanical strength and that the bores are not clogged during transdermal drug delivery. Even though, a number of fabrication techniques have been highlighted earlier, there is a need to continue research that will lead to a “gold standard” hollow microneedle drug delivery system.

## 3. Solid Microneedles

**Figure 2:**
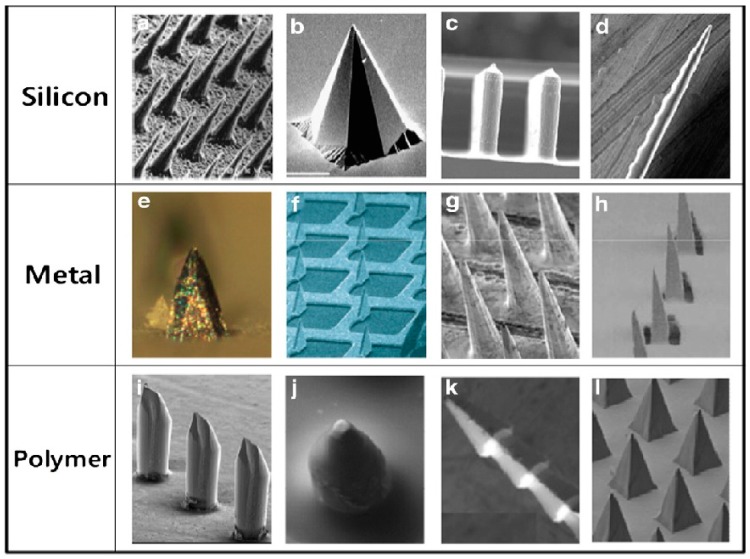
Solid microneedles fabricated from silicon, metal and polymer (Reprinted from [14] with permission. Copyright 2012 Elsevier).

Solid MN (Figure 2) deliver drugs via passive diffusion by creating microchannels to increase skin permeability followed by the application of a drug-loaded patch on the channels [26]. From a safety perspective, it is desirable for the microchannels to close soon after needle removal to prevent permeation of undesired toxic substances or infection by pathogenic microorganisms [26]. Henry *et al.* used a deep reactive ion etching process to fabricate silicon MN [27]. A chromium masking material was first deposited onto silicon wafers and patterned into dots which had a diameter approximately equal to the base of the desired microneedles. The wafers were then loaded into a reactive ion etcher and subjected to plasma etching. The regions protected by the metal mask remained to form the microneedles [27]. Vinayakumar *et al.* fabricated an array of rectangular cup shaped silicon microneedles [28]. These microneedles have the potential for reduced drug leakage resulting in improvement of drug delivery efficiency and the possibility of introducing multiple drugs [28]. The fabricated solid MN with rectangular cup shaped tip are 200 μm in height [28]. The cup shaped tips have dimensions of 60 × 60 μm (length × breadth) with a depth of 60 μm. The cups are filled with drug using a novel drop coating system [28]. Gupta fabricated stainless steel solid MN by laser cutting stainless steel sheets [26]. The desired microneedle shape and dimensions were first drafted in AutoCAD software [29]. Infrared laser was operated at 1000 Hz, 20 J/cm^2^ energy density and 40% attenuation of laser energy to cut the microneedles. A total of three passes were required to completely cut through the stainless steel sheet. MN were either prepared as individual rows of needles (“in-plane” needles) or as two-dimensional arrays of needles cut into the plane of the stainless steel sheet and subsequently bent at 90° out of the plane (“out-of-plane” needles) [29]. The stainless steel MN arrays were manually cleaned with detergent. To prepare “out-of-plane” MN, stainless steel MN were first manually pushed out of the sheet using either forceps or a hypodermic needle and then bent at 90° angle with the aid of a single-edged razor blade. Stainless steel microneedles can also be made by wet-etching photolithographically defined needle structures from stainless steel sheets [30]. The authors used these MN to deliver a vaccine based on virus-like particles containing influenza virus heterologous M2 extracellular (M2e) domains (M2e5xVLPs).

Solid MN can be fabricated from polymers. Olatunji *et al.* prepared MN from biopolymer films extracted from fish scales of tilapia (*Oreochromiss* sp.) using micromolding technique. The MN were successfully inserted into porcine skin and were shown to dissolve gradually at 0, 60, 120 and 180 s after insertion [31]. The microneedles contained methylene blue as model drug and successfully pierced porcine skin [31].

A microneedle roller is a device that contains MN mounted on cylindrical surface that rolls over the skin [6]. In this scenario, MN are mounted on a cylindrical surface and can be rolled across the skin, such that each microneedle may pierce the skin multiple times as the roller rotates across the skin [23]. In our laboratory, we studied the transdermal delivery of verapamil hydrochloride and amlodipine besylate using MN rollers and stainless steel MN. Adminpatch stainless steel microneedle arrays manufactured by NanoBioSciences™ were also used in these experiments. *In vitro* studies were performed with microneedle-treated porcine ear skin using vertical static Franz diffusion cells. Passive diffusion across untreated porcine skin served as control. Aliquots were taken every two hours for 12 h and analyzed by liquid chromatography–mass spectrometry. Transcutaneous flux of verapamil increased significantly from 8.75 to 49.96 µg/cm^2^/h across microneedle-roller treated porcine skin [13]. Percutaneous flux of amlodipine besylate following the use of stainless steel microneedles was 22.39 µg/cm^2^/h. Passive flux for the drug was 1.57 µg/cm^2^/h. This enhancement of amlodipine flux was statistically significant. Transdermal flux of amlodipine with MN roller was 1.05 µg/cm^2^/h in comparison with passive flux of 0.19 µg/cm^2^/h. The difference in flux values was also statistically significant [13].

In another study, we used stainless steel microneedle arrays (750 μm) made by Microneedle Systems^®^ to facilitate the delivery of captopril and metoprolol tartrate across porcine skin. We studied the influence of MN rollers on the diffusion of these drugs [32]. *In vitro* diffusion studies were carried out using vertical Franz diffusion cells. Transdermal flux values for captopril and metoprolol tartrate increased from 75.04 to 608.2 μg/cm^2^/h and 62.28 to 290.93 μg/cm^2^/h respectively following the use of microneedle arrays [32]. Transcutaneous flux for captopril increased from 19.68 to 1485.20 μg/cm^2^/h, whereas metoprolol tartrate flux increased from 84.64 to 226.08 μg/cm^2^/h after treatment of pig skin with microneedle rollers. There was statistically significant enhancement in transdermal flux (*p* < 0.05) of captopril and metoprolol tartrate after application of microneedle arrays and rollers [32].

Nalluri *et al.* studied the influence of AdminPatch^®^ microneedle arrays (MN) (0.6, 0.9, 1.2 and 1.5 mm lengths) and Dermaroller^®^ microneedle rollers (DR) (0.5 and 1 mm lengths) on transdermal permeation of sumatriptan [33]. A significant increase in cumulative amount of sumatriptan (SMT) permeated, steady state flux, permeability coefficient and diffusion coefficient values were observed after microneedle treatment, and the values were in the order of 1.5 mm MN > 1.2 mm MN > 0.9 mm MN > 1 mm DR > 0.6 mm MN > 0.5 mm DR > passive permeation [33]. Lag times were significantly shorter after longer microneedle application (0.24 h for 1.5 mm MN). Arrays were found to be superior to rollers with similar microneedle lengths in enhancing SMT permeation. The authors attributed this higher flux enhancement to higher density of microneedles and force of application onto skin. The *in vitro* flux values revealed that a 2.5 cm^2^ patch was adequate for effective therapy after treatment of skin with 1.5 mm MN.

Solid MN are also promising for the delivery of vaccines. Microneedle-mediated delivery of vaccines can lead to longer-lasting and more-robust antibody responses in comparison with intramuscular delivery [8]. Ultimately, this leads to improved vaccine efficacy. MN patches contain micron-scale (<1000 µm) solid needles containing a dry formulation of a vaccine that rapidly dissolves upon patch application [34]. A population immunity of approximately 93%–95% is required to interrupt measles virus transmission due to the virulence of the measles virus [34]. Edens used dissolving MN to delivery 1000 tissue culture infectious dose (TCID_50_) of measles vaccine [34]. The MN patch contained 100 pyramidal MN, each measuring 600 µm long, 300 µm wide at the base and tapering to a tip radius of less than 3 µm [34]. Following the use of this MN patch, all of the animals sero-converted and had a titer of >120 mIU/mL, which is considered protective in humans [34].

## 4. Dissolving Microneedles

Interest in dissolving microneedles is high because of a number of advantages. These include the one-step application process which is convenient for patients. Dissolving MN are fabricated on the basis of the “poke and release” principle. They are made from polysaccharides or other polymers. These MN release encapsulated drug into the skin following application and dissolution. Micromoulding is the preferred fabrication method for making dissolving MN. Certain drugs and vaccines are thermolabile so moulds are often filled with solutions of drugs and excipients and then dried under mild conditions. The fabrication process involves pouring the polymer solution into female molds, filling the microcavities of the mould under vacuum or pressure, drying under ambient conditions, centrifugation or pressure [35,36,37].

Master structures for MN supporting arrays, and pressing tools were created by Chen *et al.* using proprietary electro-discharge-machining technology [38]. Each master structure consisted of 64 (8 × 8) microstructures. Polydimethylsiloxane (PDMS) molds were created as exact inverse-replicates of the master structures. To prevent adhesion to PDMS molds, all master structures were sputter-coated with platinum [38]. PDMS molds were fabricated by pouring PDMS solution over the master structure and allowing the polymer to cure overnight at room temperature. The cured PDMS molds were then peeled from the master structures and used to make the chitosan microneedles, polylactic acid supporting arrays, and polycaprolactone pressing tools [38].

Thermal drawing is a commonly used prototyping method that can be used to make microneedle (MN) structures with ultra-high aspect ratio. However, it is difficult to repeatedly produce MNs with identical shapes using thermal drawing due to fluctuations in temperatures, drawing speeds or drawing heights [39]. Lee *et al.* fabricated master molds by thermal drawing and replicated high aspect ratio silk fibroin microneedles from these master molds multiple times [39]. The authors used poly(lactic-*co*-glycolic acid) to fabricate MN masters for micromolding. A spatially-discrete thermal drawing system was used to fabricate PLGA MN masters. Polydimethylsiloxane was used with the mixing ratio of 10:1 between silicone elastomeric base and curing agent to fabricate negative molds from the PLGA masters [39]. An aqueous silk fibroin (SF) solution from *Bombyx mori* silk worm cocoon was used to make the microneedles. To fabricate MN-shaped negative molds, PDMS resin was poured on the PLGA MN masters and cured at a room temperature for 24 h. After curing, negative PDMS molds were prepared. Finally, an aqueous SF solution was poured on the negative PDMS molds and degassed several times by application of vacuum conditions followed by drying at room temperature for 24 h.

Liu *et al.* fabricated hyaluronic acid (HA) microneedles using micromoulding [40]. Blue dye or fluorescein isothiocyanate-labeled dextran (4 kD; FD4) solution was added to 15% HA solution. HA solution containing blue dye or FD4 was placed on a micromould at room temperature. After drying for 2 h in desiccator, a polyethylene terephthalate (PET) adhesive tape was attached on the base plate for reinforcing, then 20% HA solution was placed on the PET. After drying completely, a sheet of microneedle arrays containing blue dye or FD4 was obtained by peeling the mold off [40]. The authors showed that the cumulative permeated amount of FD4 significantly increased across the skin after the application of HA microneedle arrays compared to a solution over a period of 7 h. The cumulative permeated amount of FD4 in solution was low, with a value of 0.38 ± 0.20 µg/cm^2^. In contrast, the cumulative permeated amount of FD4 increased significantly after application of microneedle arrays with a value of 231.80 ± 28.76 μg/cm^2^ over the same period. The authors observed almost no lag time in drug permeation when microneedle arrays were used [40].

An interesting and useful approach is the use of near-infrared (NIR) light-responsive polymer-nanostructure composite microneedles used for on-demand transdermal drug delivery [41]. Silica-coated lanthanum hexaboride (LaB_6_@SiO_2_) nanostructures were incorporated into polycaprolactone microneedles, serving as a near infrared ray absorber absorber [41]. When the microneedles were irradiated with NIR light, light-to-heat transduction mediated by the LaB_6_@SiO_2_ nanostructures caused the microneedle to melt at 50 °C [41].The advantage of this approach is that the release of active pharmaceutical ingredients can be triggered externally using near infrared rays.

Chen *et al.* also prepared a microneedle transdermal delivery system, composed of embeddable chitosan microneedles and a poly(l-lactide-*co*-d,l-lactide) (PLA) supporting array and used the microneedles for complete and sustained delivery of encapsulated antigens to the skin [38]. Chitosan microneedles were mounted to the top of a strong PLA supporting array, providing mechanical strength to fully insert the microneedles into the skin. When inserted into rat skin *in vivo*, chitosan microneedles successfully separated from the supporting array and were left within the skin for sustained drug delivery without requiring a transdermal patch [38].

Transdermal delivery of insulin is still a considerable challenge in the scientific community due to the low permeation rate of the drug across the skin and the difficulty of achieving therapeutic concentration with this mode of administration. Chen *et al.* combined nanovesicles with iontophoresis and used these techniques to drive insulin across microneedle-treated pig skin [42]. The penetration rate of insulin from nanovesicles driven by iontophoresis through skin with microneedle-induced microchannels was 713.3 times higher than that of its passive diffusion [42].

DNA vaccinations rely mainly on cellular delivery of plasmid DNA to induce expression of an encoded antigen, and thus lack of a proper delivery system for DNA vaccines is considered to be one of reasons for their low level of immunity [43]. A polyplex is a complex of a polymer and DNA, which is designed to protect the DNA during delivery. DNA polyplexes are efficiently protected from enzymatic degradation and exhibit increased cell transfection efficiency. MNs coated with naked DNA vaccines can induce significant antibody- and cell-mediated immune responses, thereby providing protective immunity. Kim *et al.* used microneedle arrays coated with a pH-responsive polyelectrolyte multilayer assembly (PMA) to deliver polyplex-based DNA vaccines [43].

Pearton *et al.* used coated microneedles to delivery plasmid DNA [44]. Optimization of a dip-coating method enabled significant increases in the loading capacity, up to 100 μg of plasmid DNA (pDNA) per 5-microneedle array. Coated microneedles were able to reproducibly perforate human skin at low (less than 1N) insertion forces [44]. The physical stability of the coated pDNA was partially compromised on storage, although this was improved through the addition of saccharide excipients without detriment to the biological functionality of pDNA. The pDNA-coated microneedles facilitated reporter gene expression in viable human skin [44].

## 5. Coated Microneedles

Coated microneedles refer to microneedles which are coated with the drug-containing dispersion. A plethora of techniques has been used in the literature to prepare coated microneedles. An approach using electrohydrodynamic atomisation (EHDA) principles in the preparation of smart microneedle coatings was reported in the literature [45]. Stainless steel (600–900 µm in height) microneedles were coupled to a ground electrode (in the EHDA coating set-up) with the deposition distance and collecting methodology varied for an ethanol:methanol (50:50) vehicle system. The authors used this technique to prepare nano- and micrometer-scaled pharmaceutical coatings [45]. Fluorescein dye (serving as potential drug, sensory materials or disease state markers) and polyvinylpyrrolidone (PVP, polymer matrix system) formed the remaining components of the coating formulation. Based on these excipients and by varying the coating process, particles (100 nm to 3 µm) and fibres (400 nm to 1 µm) were deposited directly on MNs in controlled and selectable fashion [45].

Ma and Gill used a polyethylene glycol matrix containing a water insoluble drug lidocaine to coat solid microneedles [46]. Uniform coatings were obtained on microneedle surfaces. *In vitro* dissolution studies in porcine skin showed that microneedles coated with PEG-lidocaine dispersions resulted in significantly higher delivery of lidocaine in just 3 min compared with 1 h topical application of 0.15 g EMLA^®^, a commercial lidocaine-prilocaine cream [46].

## 6. Hydrogel-Forming Microneedles

The first two microneedle-based products, just recently marketed, Soluvia^®^ and Micronjet^®^, are based on metal and silicon, respectively [47]. However, the current trend in microneedle-based research has recognized biocompatibility problems associated with the use of silicon and the potential for inappropriate re-use of silicon or metal microneedles, which remain fully intact after removal from a patient’s skin. This has led to a multiplicity of technologies aimed at overcoming this shortcoming. In this regard, recent effort has focused on microneedles formulated from aqueous polymer gels. One of these approaches involves the use of hydrogel-forming microneedles [47]. One of the differences and advantages in comparison with regular dissolving polymer microneedles is that by using this drug delivery system, delivered doses of drugs and biomolecules are no longer limited to what can be loaded into the needles themselves.

Donnelly *et al.* prepared hydrogel-forming microneedles from “super-swelling” polymeric compositions. These are microneedle arrays, prepared under ambient conditions, which contain no drug themselves [48]. Instead, they rapidly imbibe skin interstitial fluid upon insertion to form continuous conduits between the dermal microcirculation and an attached patch-type drug reservoir [48]. Such microneedles act initially as a tool to penetrate the stratum corneum barrier. Upon swelling, they become a rate controlling membrane. Fluid uptake range in one hour was 0.9–2.7 μL which is of the same order of magnitude as the rates of interstitial fluid uptake for hollow microneedles and microdialysis [49]. Other advantages of hydrogel-forming microneedles are that they can be fabricated in a wide range of patch sizes and geometries, can be easily sterilized, resist hole closure while in place and are removed completely intact from the skin [50]. While these systems may work for high-potency, low dose drugs and vaccines, there is a need to broaden their application to include medications that are administered for the management of chronic disorders. Recently, there was a report on the investigation of various polymeric compositions in order to find materials capable of rapid swelling, but which would be sufficiently hard in the dry state to penetrate the skin. These materials, once swollen, should maintain structural integrity and be reasonably robust during handling [48]. Stock solutions of Gantrez S-97 (40% *w*/*w*) or AN-139 (30% *w*/*w*) were prepared using deionized water. Hydrogel films were then prepared using varying concentrations of the co-polymer, PEG 10,000 and the modifying agent, sodium carbonate. Films were cured at 80 °C for 24 h to induce chemical crosslinking between the PMVE/MA and PEG by ester formation. Swelling studies were conducted and the results used to select optimal polymeric compositions [48]. A microneedle formulation with enhanced swelling capabilities from aqueous blends containing 20% *w*/*w* Gantrez S-97, 7.5% *w*/*w* PEG 10,000 and 3% *w*/*w* Na_2_CO_3_ and using a drug reservoir of a lyophilized wafer-like design was selected. These microneedle-lyophilised wafer compositions were robust and effectively penetrated skin, swelling extensively, but they were removed intact [48]. Significantly, *in vitro* delivery experiments across excised neonatal porcine skin showed that approximately 44 mg of the model high dose small molecule drug ibuprofen sodium was delivered in 24 h, equating to 37% of the loading in the lyophilised reservoir. The super swelling microneedles delivered approximately 1.24 mg of the model protein ovalbumin over 24 h, equivalent to a delivery efficiency of approximately 49% [48]. Silicon MN arrays to be used as master templates in micromoulding of hydrogel-forming arrays were first microfabricated. Using the above silicon MN arrays as master templates, silicone elastomer micromoulds were then prepared. The authors fabricated microneedles with enhanced swelling capabilities from aqueous blends containing 20% *w*/*w* Gantrez S-97, 7.5% *w*/*w* PEG 10,000 and 3% *w*/*w* Na_2_CO_3_ and utilized a drug reservoir of a lyophilized wafer-like design. In *in vitro* delivery experiments across excised neonatal porcine skin, approximately 44 mg of ibuprofen sodium was delivered in 24 h, equating to 37% of the loading in the lyophilized reservoir. The hydrogel-forming microneedles also delivered approximately 1.24 mg of the model protein ovalbumin over 24 h, equivalent to a delivery efficiency of approximately 49% [48].

In a recent report, investigators incorporated caffeine and lidocaine hydrochloride into transdermal microneedle systems using novel laser-engineered polymers. Potential pediatric applications were studied [51]. The authors prepared adhesive patches using aqueous blends consisting of 10% *w*/*w* PMVE/MA and 5% *w*/*w* tripropyleneglycol methyl ether [51]. Facilitated transdermal transport of caffeine and lidocaine HCl were observed using drug-loaded dissolving MN and integrated hydrogel-forming MN in comparison to their corresponding control patches/cream across dermatomed skin over a period of 24 h [51].

The use of hydrogel-forming MN is promising. Indeed more effort should be directed at widening the range of materials that can be used to fabricate these useful transdermal drug delivery systems. Although hydrogel-forming MN are made from polymers, there are distinct differences between the two groups of MN. The possibility of incorporating active pharmaceutical ingredients in a separate reservoir means that it is possible to increase the amount of delivered medications.

## 7. Clinical Trials

In 2009, a six-month, randomized, multicenter, blinded, multi-dose, Phase 2 clinical study using MN to deliver recombinant human parathyroid hormone 1-34, teriparatide (PTH, 4.1 kDa) was carried out [52]. The study demonstrated that 40 µg of PTH delivered using solid titanium MN was capable of delivering an effective amount of PTH, comparable to a 20 µg of Forteo (PTH) injection. Zosano Pharma Corporation has developed ZP-PTH which is a transdermal MN formulation of parathyroid hormone 1-34 (PTH) [52,53]. The product has completed Phase 2 clinical trials and is scheduled for Phase III trials. If approved, it will be used for the management of osteoporosis. The product is based on titanium microneedle arrays produced by photochemical etching. The MN come with a reusable applicator. NanoPass Technologies also has an intellectual property-backed product called MicronJet™ [52]. It is a single use, microneedle-based device for intradermal delivery of drugs, proteins and vaccines.

## 8. Challenges

There is a tremendous amount of research being carried out to study the influence of MN on transdermal drug delivery. There is immense potential for the use of these micron-sized needles for transdermal drug delivery enhancement. However, for these needles to find widespread clinical applications, a number of challenges also have to be addressed including irritation, microbial contamination and the delivery of therapeutically relevant concentrations of drugs. There is also a limited choice of appropriate biomaterials, lack of mechanical strength, poor control of drug delivery, and limitation of drug loading dose [39]. Potent drugs requiring low doses and vaccines seem to be the drugs most likely to be delivered in therapeutically useful concentrations. Another challenge is the delivery of macromolecules- products of biotechnology. These molecules have high molecular weights and also high hydrophilicity making them particularly challenging to deliver across the skin. There is also the concern that some MN such as those fabricated from silicon and some polymers may not have adequate mechanical strength to pierce the skin. The ideal scenario is to fabricate MN with a low insertion force and a high fracture force. It is also cumbersome for MN to be applied in a two-step manner, that is to porate the skin first and then apply a patch. In this regard, the use of hydrogel-forming microneedles is particularly interesting. There is also the need to balance penetration enhancement with painlessness.

## 9. Conclusions

This paper has described some of the landmark achievements in MN fabrication and applications. Several important milestones have been achieved through the use of micro-electro-mechanical systems (MEMS) technologies and those developed specifically to optimize MN for transdermal drug delivery. Valid concerns regarding the use of these MN especially safety issues have also been highlighted. There are concerns about delayed onset of action, costs of the delivery system, possible accidental use, misuse or abuse [51]. There is a need to investigate further skin pore closure after MN application especially as it relates to the risk of infections. It is also essential to ensure that materials which are used for MN fabrication do not induce skin irritation. Another area is the need for a balance between increased permeability and painlessness. It is known that as MN length increases, there is a high probability that pain receptors located in the dermis may be stimulated. In spite of the above-mentioned limitations, the outlook for the use of these devices is promising even as more work needs to be done for microneedles to become routine drug delivery systems in clinical practice.
